# Nanotechnological Applications Based on Bacterial Encapsulins

**DOI:** 10.3390/nano11061467

**Published:** 2021-06-01

**Authors:** Javier M. Rodríguez, Carolina Allende-Ballestero, Jeroen J. L. M. Cornelissen, José R. Castón

**Affiliations:** 1 Department of Structure of Macromolecules, Centro Nacional de Biotecnología (CNB-CSIC), Campus de Cantoblanco, 28049 Madrid, Spain; jmrodriguez@cnb.csic.es (J.M.R.); carol.allende@hotmail.com (C.A.-B.); 2 Department of Molecules and Materials, MESA+ Institute for Nanotechnology, University of Twente, 7500 AE Enschede, The Netherlands; j.j.l.m.cornelissen@utwente.nl; 3 Nanobiotechnology Associated Unit CNB-CSIC-IMDEA, Campus Cantoblanco, 28049 Madrid, Spain

**Keywords:** encapsulins, nanocompartments, encapsulated enzymes, encapsulin-based-nanotechnology

## Abstract

Encapsulins are proteinaceous nanocontainers, constructed by a single species of shell protein that self-assemble into 20–40 nm icosahedral particles. Encapsulins are structurally similar to the capsids of viruses of the HK97-like lineage, to which they are evolutionarily related. Nearly all these nanocontainers encase a single oligomeric protein that defines the physiological role of the complex, although a few encapsulate several activities within a single particle. Encapsulins are abundant in bacteria and archaea, in which they participate in regulation of oxidative stress, detoxification, and homeostasis of key chemical elements. These nanocontainers are physically robust, contain numerous pores that permit metabolite flux through the shell, and are very tolerant of genetic manipulation. There are natural mechanisms for efficient functionalization of the outer and inner shell surfaces, and for the in vivo and in vitro internalization of heterologous proteins. These characteristics render encapsulin an excellent platform for the development of biotechnological applications. Here we provide an overview of current knowledge of encapsulin systems, summarize the remarkable toolbox developed by researchers in this field, and discuss recent advances in the biomedical and bioengineering applications of encapsulins.

## 1. Introduction

Cells acquire more precise control over their metabolism by subcellular compartmentalization of closely related processes in organelles, subcellular structures with a defined boundary layer that encloses a proteomically distinct interior [1]. Complex, membrane-based organelles were historically considered a distinctive characteristic of eukaryotic cells. Enabled mainly by advances in imaging techniques, recent decades have witnessed a steady increase in our understanding of the complexity of the subcellular architecture of some bacterial and archaeal species [2,3,4]. Subcellular compartments delimited by semi-permeable protein shells [5,6], lipid bilayers [7,8], lipid monolayers [1], or phase-separated membraneless condensates [9] are now being described with increased detail.

Among the prokaryotic protein-bounded compartments are the bacterial microcompartments (BMC) [10,11] and the encapsulins [5,12,13]; these micro- and nanometer-sized semi-permeable protein cages encase an enzymatic core. BMC are complex polyhedral structures, ~40 to 200 nm in diameter, built of many thousands of protein subunits of 10–20 distinct types [14]. They are encoded in genetic modules (BMC loci) in which the components necessary for assembly of a functional BMC are organized in a compact structure. BMC loci are widely distributed in bacteria and have been identified in 45 phyla [11]. To increase reaction efficiency, complete segments of metabolic pathways are encapsulated within BMC, including anabolic (carboxysomes; [15]) and catabolic reactions (metabolosomes; [14]).

Carboxysomes are found in all cyanobacteria and some chemoautotrophs; they contain the enzymes carbonic anhydrase (CA) and D-ribulose-1,5-bisphosphate carboxylase/oxygenase (RuBisCO). RuBisCo, which catalyzes the first step in CO_2_ assimilation into the biosphere, is a key enzyme for life on Earth, although its poor affinity for CO_2_, low catalytic rate, and use of O_2_ as an alternative substrate make RuBisCo notoriously inefficient [16]. Carboxysomes are a successful strategy evolved by photosynthetic organisms to overcome RuBisCo limitations. The carboxysome shell allows cytosolic bicarbonate entry into the lumen of the compartment, which is filled with CA and RuBisCo in close proximity. CA catalyzes bicarbonate to produce CO_2_, which is retained in the lumen by the selective permeability of the shell [17,18]. The shell also excludes O_2_ from the carboxysome lumen [15]. As a result, CO_2_ reaches high concentrations in the carboxysome lumen, and RuBisCo rate and specificity increase.

Improved understanding of the molecular structures and underlying mechanisms evolved to optimize biochemical reactions has stimulated molecular engineers to modify bacterial protein compartments [5,10] and other synthetic and natural protein cages for biotechnological applications [19]; these include lumazine synthase [20], ferritins [21], virus-like particles [22], and vault ribonucleoproteins [23]. Although protein cage engineering is in its infancy, we are already witnessing the development of promising applications in fields as diverse as vaccine therapy [24], drug delivery [25], medical imaging [26], photodynamic treatments for cancer [27], and pest control [28].

This review presents an overview of the structural and functional features of encapsulins, outlines the remarkable toolbox developed by researchers, and highlights recent advances in biomedical and bioengineering applications of encapsulins.

## 2. Encapsulins

The encapsulins are a family of ~30 kDa bacterial and archaeal proteins that self-assemble into icosahedral compartments which range in size from 20 to 42 nm. These nanocompartments are similar to virus capsids in the icosahedral architecture of the container as well as in the protomer fold, which is structurally similar to capsid proteins of the Hong Kong 97 (HK97)-like virus lineage that includes the Caudovirales bacteriophages [6,29,30,31,32].

**Figure 1:**
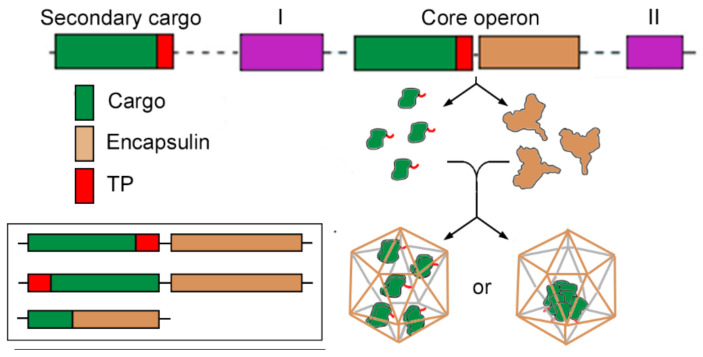
Organization of the encapsulin system. The core operon includes two genes, a cargo gene (green) and an encapsulin gene (orange). The targeting peptide (TP, red) is at the cargo C-terminus, but could be located at the N-terminus or be absent (inset). Other components associated to the operon without TP are indicated as I and II (purple). Secondary cargos are not included in the core operon but contain a TP. The cargo monomers are encapsulated as small oligomers at a high copy number (**left**) or associated into one or two large oligomers (**right**).

Much like BMC, encapsulin nanocompartments are proteinaceous shells that enclose an enzyme core. Whereas BMC are highly complex structures encoded by a large set of genes, simplicity is the hallmark of encapsulins [6]. Most encapsulin systems are encoded in a two-gene operon, in which one gene encodes the protomer that self-assembles into the icosahedral nanocompartment, and the other encodes an enzymatic multimeric cargo. Cargo enzymes are targeted to the nanocompartment lumen via a cargo-loading peptide (CLP) or targeting peptide (TP) located at their termini, which interacts non-covalently with a binding pocket on the shell luminal surface [6,33,34]. Most archaeal systems have an even more compact configuration in which shell and cargo are fused, and a single gene encodes a completely functional nanocompartment [6,29,32,35,36] (Figure 1).

Both types of compartments, BMC and encapsulins, are thought to confer similar benefits on microorganisms [10,35]. Separation of the encapsulated enzymatic core from the cytosol protects the cell from toxic metabolic intermediates, prevents unwanted side reactions, provides optimal concentration of metabolites and enzymes in multi-step reactions and, in the case of encapsulins, storage for key metals in the lumen of the nanocontainer.

Encapsulins were originally characterized as 20–30 nm-diameter, high molecular weight proteinaceous particles composed of a 32 kDa single subunit protein, abundant in supernatants of *Brevibacterium linens* M18, with broad-spectrum bacteriostatic activity [37]. Homologs with diverse activities were characterized in other bacterial species [38,39,40,41,42,43]. The high-resolution structure of the archaeon *Pyrococcus furiosus*, initially thought to be a virus-like particle, was the first encapsulin atomic structure determined [29]. It was not until seminal work with the *Thermotoga maritima* encapsulin [6] that the true nature of encapsulins was understood.

There has been a gradual improvement in the algorithms for sequence similarity database searches and a continuous increase in the size and diversity of sequenced genomes of bacteria and archaeal microorganisms; this is producing a progressively sharper picture of the true abundance and diversity of encapsulins in nature [6,29,31,35]. The most recent computational database search identified over 6000 encapsulin-like systems in 31 bacterial and four archaeal phyla, grouped in four different families based on particle structure [32]. Similar analysis of a small fraction of the environmental metagenomic data available uncovered new encapsulins [44], which suggests that other encapsulin systems await discovery, and that these systems constitute a widespread prokaryotic compartmentalization strategy.

The evolutionary origin of encapsulins remains elusive; this is due in part to the low sequence similarity between encapsulins and members of the HK97-like virus lineage, which implies very old origin, and in part to the few atomic structures available. Based on phylogenetic and structural analyses, encapsulins are proposed to have a viral origin, possibly via “domestication” events of prophage HK97-type capsid proteins [6,29,30,31,32]. Eukaryotic cells show evidence of similar events involving the Ty3/gypsy superfamily of retrotransposons, ancient mobile elements that are widely distributed and often abundant in their genomes, considered ancestral to modern retroviruses [45]. This and other retroviral-like elements have repeatedly been repurposed for cellular functions throughout evolution [46]. An origin strikingly similar to that of encapsulins has been suggested for the bacterial phage tail-like bacteriocins, or tailocins. These are very large protein assemblies (2–10 MDa) structurally related to various phage tails, whose function is to kill competing bacteria [47]. They are widespread and diverse among bacteria, and some have diverged considerably, which suggests they could be quite ancient [48].

Encapsulins are prevalent in bacterial and archaeal hosts that occupy extreme habitats but have been identified in microbes that inhabit practically all terrestrial and aquatic habitats, and are also found in bacterial pathogens [32,35]. It is proposed that, in a broad sense, encapsulin systems might be a general strategy in microbes to sequester toxic transformations and molecules, and act as specific storage compartments [35].

### 2.1. Nanocompartment Structure

Structural analysis of encapsulins is still limited to a few cases. At present, eight encapsulins have been resolved at near-atomic resolution by X-ray crystallography or three-dimensional cryo-electron microscopy (3D cryo-EM). These are the encapsulins from the archaeon *P. furiosus* (EncPf; PDB entry 2e0z) [29], and from the bacteria *T. maritima* (EncTm; PDB entry 3dkt) [6], *Myxococcus xanthus* (EncMx; PDB entry 4pt2) [30], *Quasibacillus thermotolerans* (EncQt; PDB entry 6nj8) [49], *Synechococcus elongatus* (EncSe; PDB entry 6 × 8 m) [31], *Mycolicibacterium hassiacum* (EncMh; PDB entry 6i9g) [50], *Mycobacterium smegmatis* (EncMs; PDB entry 7boj) [51], and *Haliangium ochraceum* (EncHo) [52]. All these encapsulins except EncSe are close relatives and are grouped in the classical family 1; EncSe belongs to family 2A and has distinctive structural and functional features. In addition, the 3D structure of the *B. linens* encapsulin was determined at low resolution by cryo-EM analysis (EncBl; Electron microscopy accession no. EMD-3608) [53]. An adapted shotgun electron microscopy method identified numerous protein complexes in *M. smegmatis* and were used efficiently to find new encapsulin nanocages [54].

**Figure 2:**
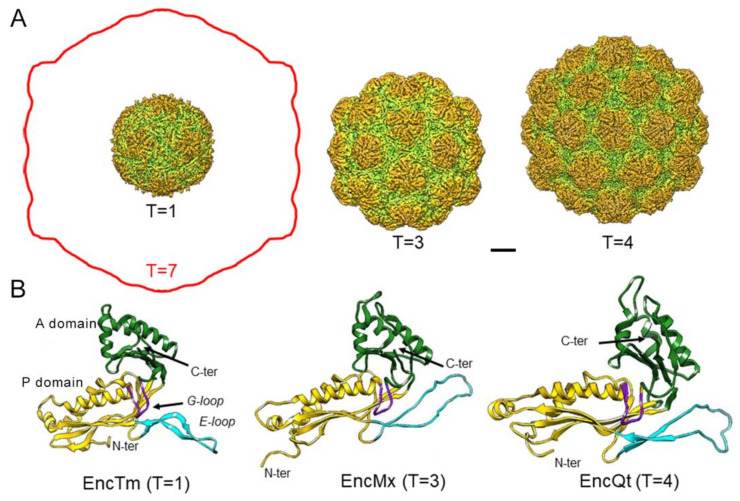
Structure of encapsulin nanocompartments. (**A**) Encapsulin nanocompartment size increases with the triangulation number (T), from (left to right) *T. maritima* (EncTm; PDB entry 3dkt) with a T = 1, *M. xanthus* (EncMx; EMD accession code 5917) with a T = 3 to *Q. thermotolerans* (EncQt; EMD accession code 9383) with a T = 4 shell. For comparison, the contour of the HK97 bacteriophage capsid with a T = 7 lattice is outlined (red). Bar, 50 Å. (**B**) Ribbon diagrams of EncTm (PDB entry 3dkt), EncMx (PDB entry 4pt2), and EncQt (PDB entry 6nj8). The N and C termini are indicated. The encapsulin fold has three major domains: A domain (green), P domain (yellow), and the E-loop (cyan). The G-loop is indicated (purple).

Like viruses with icosahedral symmetry, encapsulins assemble into icosahedral shells (as described by the concept of quasi-equivalence [55]), and are defined by their triangulation number (T). The simplest encapsulin nanocompartments are built from 60 identical subunits assembled into 12 pentamers with a T = 1. EncTm, EncSe, EncMh, and EncBl are found naturally as 20–24 nm-diameter T = 1 particles. Encapsulin particles with more than 60 identical subunits assemble into pentamers and hexamers (T > 1), and subunits cannot have identical, but only quasi-equivalent environments in the shell. Quasi-equivalence allows assembly of larger icosahedral particles, but only with certain values of T [56]. Thus far, encapsulins larger than T = 1 have T values of 3 (such as EncPf and EncMx, 30–32 nm in diameter) or 4 (such as EncQt, 42 nm) (Figure 2A).

Despite their low sequence similarity, comparison of the encapsulin fold and the capsid protein fold of HK97-like virions (the major capsid protein gp5 of the HK97 virus [57] shows high structural similarity, which indicates that both compartments descend from a common ancestor [58]. The HK97-like viruses are the most successful self-replicating system on earth [59]; they include the head-tailed phages, tailed haloarchaeal viruses, and herpesviruses, all of which infect hosts from all domains. Capsids with the HK97-like core (also termed Johnson fold) can have larger T numbers to accommodate a large viral genome, from T = 7 (such as bacteriophages HK97, T7, P22 and λ, 60–70 nm diameter) to T = 52 (such as jumbophage G, 185 nm diameter) [60]. To control assembly of these complex capsids, one or more auxiliary proteins such as a scaffolding protein are nonetheless usually needed. Encapsulins might have originated from an ancestral capsid protein after accumulation of mutations that resulted in less sophisticated capsids; these simple assemblies progressively lost the need for a scaffolding protein, protease-mediated maturation, as well as genome packaging machinery. Alternatively, an ancestral simple cellular encapsulin might have incorporated additional elements to render complex viral capsids.

Encapsulin subunits are built of three domains: the peripheral (P) domain, the axial (A) domain, and the elongated (E-) loop. Additional structural elements might include an N-terminal region (as in EncSe) and a G-loop (Figure 2B). A and P domains are similar among encapsulin structures; the triangular A domain forms the five- and pseudo sixfold symmetry axis interface contacts, and the P domain is at the periphery of pentamers and hexamers. The E-loop might acquire an extended conformation as in EncTm, EncMh, EncMs and EncHo or, after a 60–70° rotation, a compact conformation as in EncMx, EncPf, EncSe and EncQt, similar to that of the HK97-like capsid proteins. An extended E-loop conformation allows tight contacts between the twofold symmetry-related subunits, whereas most interactions of the E-loop with the compact conformation are with adjacent monomers of the same pentamer or hexamer. This striking difference might involve different encapsulin assembly pathways, mediated by encapsulin dimers (those with an extended E-loop) or by encapsulin hexamers and pentamers (those with a compact E-loop).

Encapsulin cages have ~3 to 10 Å-diameter channels that penetrate the shell. These pores, located around the five and threefold axes and at subunit interfaces, have distinct local environments with specific distribution of positive and negative charges on their inner and outer surfaces. The pores could serve as selective channels through which substrates/products can be interchanged with the cytoplasm to access the confined enzyme cargo. In the EncQt T = 4 shell, an iron storage nanocompartment, all pores are negatively charged on the exterior and interior surfaces, which implies that they are optimized to attract and channel positively charged ions [49]. In contrast, the 5-fold axis pores of EncSe are positively charged, but Cys is the likely substrate of the enzyme cargo [31]. In EncTM, the fivefold axis pores contain two rings of His and Tyr residues, and the threefold axis pores have a ring of Phe residues that probably slow iron permeability [61].

**Figure 3:**
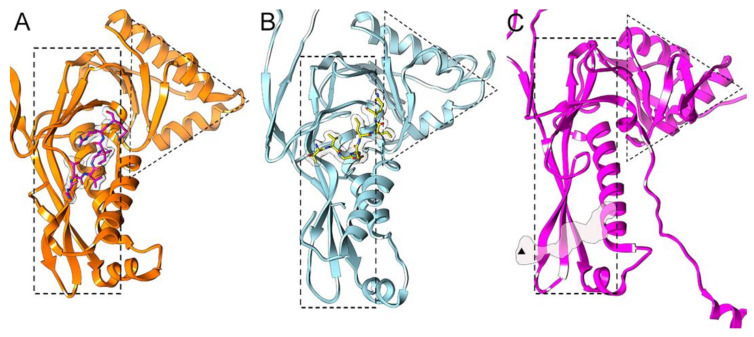
Binding pockets for the targeting peptide that directs protein cargo encapsulation. (**A**) The EncTm amphipathic binding pocket is located on the inner surface of the P domain. The TP of the enzyme EncFtn (GGDLGIRK) is shown (white density). TP residues are displayed as sticks (PDB entry 3dkt). (**B**) The pocket and binding of the TP of the IMEF cargo (TVFSLIQ) in the EncQt system is similar to that of EncTm (PDB entry 6nj8). (**C**) Binding of the TP of the cysteine desulfurase in the EncSe system (PDB entry 6 × 8m) is near the three-fold symmetry axis (black triangle), distant from the pockets in EncTm and EncQt. Dashed rectangles and triangles correspond to P and A domains, respectively.

The encapsulation mechanism of the enzyme cargo is usually mediated by a TP in the protein cargo, although protein cargo and encapsulin are fused in EncPf. The TP is necessary and sufficient to load heterologous proteins in the interior of this nanocompartment [6,62,63]. The binding pocket is located on the interior surface of the P domain, and interaction with the TP is based on shape complementarity, as well as on hydrophobic interactions and salt bridges between the two partners. Whereas the binding pocket is relatively similar in EncTm and EncQt, in EncSe this pocket is found in an adjacent region, close to the threefold symmetry axis (Figure 3). At 2 Å resolution, the EncTm structure showed a 60-flavin ligand on the shell outer surface, which suggests a direct EncTm role in iron metabolism [61].

The TP-mediated, flexible interactions between the cargo and the encapsulin shell contribute to the low resolution observed for the encapsulated cargo. In the EncMs system, however, the dye-decolorizing peroxidase (DyP) cargo was clearly resolved in the cryo-EM structure [51]. DyP forms a dodecameric complex of two hexamers that stretches across the interior of the encapsulin, which contributes to DyP stability.

Atomic force microscopy (AFM) of the bacterial nanocompartments provides information not evident from structural data, such as direct measurement of mechanical stiffness or brittleness. EncBl and EncTm analysis using AFM nanoindentation showed that these nanocompartments are similar to the rigid HK97-like bacteriophage capsids [64]. For EncBl, cargo-loaded particles (with native or non-native cargos) are less stable than empty particles, which indicates that TP binding to the shell interior locally distorts icosahedral symmetry; this distortion might be needed for optimal function of the hexameric DyP enzyme complex.

### 2.2. Encapsulin Systems

Encapsulin systems are identified by the sequence similarity of their shell proteins. The latest genomic survey found 6133 encapsulin-like proteins in the UniProtKB database. Considering the degree of this similarity, their Pfam family membership, and the genomic organization around their genetic loci, Andreas and Giessen [32] extended the number of members of the previously defined families 1 [35] and 2 [31] to 2383 and 3523, respectively, and described two new minor families, families 3 and 4 with 132 and 95 systems, respectively.

#### 2.2.1. Family 1

Family 1 encapsulin-like proteins are the most widespread systems, found in 31 of 35 prokaryotic phyla that encode encapsulin-like operons. They were the first type of encapsulin system identified and are so far the best-characterized. A model encapsulin system of this family is composed of a core operon that encodes the encapsulin shell gene, usually preceded by the core cargo protein gene [35]. Core cargo proteins are characterized by a TP (a 10–40 residue disordered region) at the C terminus. Proteins that also contain a TP not encoded in the core operon can be encapsulated and are referred to as secondary cargos (see Figure 1). Multiple secondary cargos are not unusual, and up to four different secondary cargos have been identified in a single genome [35]. In the EncMx system, the best-studied example of systems with multiple cargo proteins, the core operon is formed by the encapsulin shell gene (EncA), located downstream of the core cargo EncB gene. Two genes elsewhere in the genome encode secondary cargos EncC and EncD, which are co-encapsulated with EncB [30]. Additional proteins near the core operon, which are not able to encapsulate but are conserved, are referred to as associated components.

Within family 1, six operon types can be distinguished based on the nature of the core cargo protein. The largest, with 1505 members, contains a DyP preceding the encapsulin gene. DyP are heme-containing peroxidases, different from standard peroxidases because of their distinctive primary sequences and tertiary structures, characterized by their ability to catalyze the oxidation of phenolic and non-phenolic aromatic compounds, although their physiological function and natural substrates are not known [65]. Because peroxidases consume H_2_O_2_, they often participate in defense against oxidative stress [66]; they are found as monomers, dimers, tetramers, and hexamers in solution [67]. Encapsulated DyP are characterized by a C-terminal extension containing the TP, and form homomultimeric complexes. DyP B of *B. linens* is organized as a trimer of dimers [6,53,64] that probably interacts with the shell though the C-terminal TP of the three subunits that face the shell at positions surrounding the threefold axis pore. The encapsulated DyP of *M. smegmatis* is a dodecamer that consists of two hexamers related with a twofold axis of symmetry [51].

The second largest operon type has 594 systems, with cargo proteins of the large ‘ferritin-like superfamily’ that share all or part of a characteristic four-helix bundle structural motif [68]. Four types of cargo proteins have been identified; two of them, similar to bacterioferritins and to hemerythrins, have not been structurally characterized and will not be further discussed here. Systems that encode the other two cargo types, encapsulated ferritin-like proteins (EncFtn) and iron-mineralizing encapsulin-associated firmicute (IMEF) protein, have been partially characterized and used in different biotechnological applications. These two types of nanocompartments can biomineralize iron, analogously to classical ferritins, but on a much larger scale. The classical iron storage ferritin nanocage consists of 24 subunits configured as a polyhedron, with channels that capture and direct Fe^2+^ to the ferroxidase active sites where it is oxidated to Fe^3+^ [68]. Ferritin cages store the resulting Fe^3+^ as a ferrihydrite mineral inside the central cavity.

The discoidal decameric structure of EncFtn proteins is arranged as a pentamer of dimers [36,69]. EncFtn dimerization is mediated by two iron atoms that stabilize the dimer interface to reconstitute the four-helix ferritin fold, generating a functional ferroxidase center on this interface. In classical ferritins, the exit sites of the ferroxidase centers face the central storage space [70], whereas in EncFtn they face the opposite direction [69]. Due to this organization and to the lack of an enclosed cavity, EncFtn decamers are unable to store mineralized iron. Instead, in EncFtn-loaded nanocontainers, the oxidation process and biomineralization/storage effected by classical ferritins are divided between the EncFtn complexes, which act as ferroxidases, and the shell, which provides the iron mineralization and storage environment [69]. The nanocompartment of *M. xhantus*, which contains two EncFtn cargos in a T = 3 icosahedral shell, is able to store up to ~30,000 iron atoms in dense iron cores of ~24 nm [30].

In some Archaea encapsulin systems, a distinct type of EncFtn proteins are fused to the encapsulin capsid, which results in EncFtn domain internalization in the assembled container [6]. When expressed separately from the encapsulin, the EncFtn domain of *P. furiosus* assembles into a decameric structure as do other EncFtn cargos, although its organization within the nanocompartments has not been determined [36].

The IMEF cargo was initially described in the Firmicutes bacteria [35]. Sequence and structure analysis show that the IMEF cargo is a distinct class of ferritin-like proteins with no known ferroxidase motifs in the primary sequence; however, the IMEF cargo of *Q. thermotolerans* folds into the four-helix bundle characteristic of the ferritin-like superfamily [49]. It forms dimers in solution and when encapsulated with two Fe atoms bound at the subunit interface, generates a ferroxidase site. The *Q. thermotolerans* encapsulin that forms large T = 4 capsids, ~42 nm in diameter, can store up to ~83,000 iron atoms in ~36 nm iron-rich cores [49].

Another type of family 1 operon was identified in the anaerobic ammonium-oxidizing (anammox) bacteria of the phylum *Planctomycetes* [35]. In these systems, the encapsulin contains an N-terminal diheme cytochrome C fusion domain and is associated with a core cargo gene that encodes a nitrite reductase-hydroxylamine oxidoreductase (NIR-HAO). These systems have been implicated in the anammox process (the conversion of nitrite and ammonium ions directly into diatomic nitrogen and water), although their biological role is currently unknown [35,71].

#### 2.2.2. Family 2

The largest group of encapsulins belongs to family 2 [31], which includes 3523 members in 14 bacterial phyla [32]. Systems in this family can be separated into two general groups based on the absence or presence of an internal cyclic nucleotide-monophosphate (cNMP)-binding domain in the E-loop (families 2A or 2B, respectively) [31,32]. Encoded cargo proteins include cysteine desulfurases, terpene cyclases, polyprenyl transferases, and xylulose kinases. In most family 2A systems, the core operon has a cysteine desulfurase gene downstream of the encapsulin. In the family 2A EncSe system, an N-terminal disordered region of 225 amino acid residues is necessary for efficient encapsulation of heterologous cargo in an *E. coli* expression system [31]. With the exception of the xylulose kinases, cargo proteins in this family have long unannotated regions at their termini, which are predicted to be disordered and could act as encapsulation signals [32]. Organization of family 2B operons is complex due to the frequency in the same operon of two distinct cNMP-domain-containing encapsulin shell proteins. Since no system of this family has been characterized, however, it is not known whether these two encapsulins each assemble into a separate nanocontainer or form a single mixed shell.

#### 2.2.3. Family 3

Family 3 encapsulin-like systems were identified almost exclusively in the genomes of members of the phyla Actinobacteria and Proteobacteria [32], within natural-product biosynthetic gene clusters (BGC), e.g., genome locations where genes involved in the same pathway for synthesis of a natural compound are grouped [72,73]. Based on the characteristics of the BGC, they are classified in six distinct operon types. The nature of the putative cargo proteins is unknown, but the presence of encapsulins in BGC might indicate their involvement in the encapsulation of some enzymes on the pathways encoded. Some members of this family have a unique structure, with four to five predicted transmembrane helices in a C-terminal extension of the shell protein; this led to the suggestion that it could mediate formation of a new type of hydrophobic pore, allow interactions with lipids, or even recruit a lipid envelope [32].

#### 2.2.4. Family 4

Family 4 encapsulin-like systems are restricted to thermophilic anaerobe microorganisms isolated from submarine hydrothermal vents [32]. Their protein shell has a very distinctive structure; compared with other encapsulins, it appears to have large deletions in the amino and carboxy termini, resulting in a protein with the A domain only. As the A domain is largely responsible for interactions that stabilize the pentamers in the icosahedral capsids [59,60], these truncated encapsulin forms might produce pentamer facets or larger aggregates.

### 2.3. Cargo Loading

Cargo loading is central to the physiological role of encapsulins and, by extension, to the development of biotechnological applications. Cargo loading has been studied in some detail in family 1 systems [33,34]. Information regarding family 2 systems is limited to the analysis of EncSe, in which the encapsulation signal resides in a large N-terminal disordered region of the cargo protein and requires more than 100 amino acid residues for efficient loading [31]. Family 3 and 4 systems remain to be characterized.

In some family 1 systems, cargo is loaded through direct fusion of a functional domain with the encapsulin shell protein N terminus, which projects towards the nanocompartment lumen, leading to particles whose inner surface is lined with the fused domains. In the archaea *P. furiosus*, an EncFtn domain is fused to the encapsulin gene N terminus [29,36]. A similar strategy is found in the encapsulin systems described in the anammox bacteria of the phylum Planctomycetes [35,44], in which a cytochrome domain is fused to the N terminus. In these systems, the putative core cargo protein (NIR-HAO) lacks detectable targeting signals, and its encapsulation is thought to occur via interaction with the cytochrome domain [35].

In most family 1 systems, however, cargo is loaded via CLP; these are short peptides (~10 amino acids) at the C-terminal end of the cargo protein, separated from the native cargo domain by variably sized flexible linkers, generally rich in alanine, proline, and glycine residues. N-terminal encapsulation sequences have only been described for the ferredoxin secondary cargo, which is frequently associated with the IMEF operons in Firmicutes [35]. CLP interact with conserved pockets on the interior surface of encapsulin compartments and have strong sequence similarity [6,34,35,49]. Consensus sequences based on cargo proteins are commonly used to identify new cargo proteins [34,35]. CLP based on these consensus sequences mediate cargo encapsulation when fused to heterologous proteins, albeit less efficiently than the native sequences [34]. Cargo loading might take place during encapsulin assembly and can be emulated efficiently in vitro by adding CLP-tagged proteins to solutions of disassembled monomers [33,74,75].

Encapsulin and core cargo genes form tightly packed operons, which suggests strong translational coupling, but there are no data regarding the regulation of the expression and encapsulation of secondary cargos, which are frequently encoded in distant loci. Operons with multiple secondary cargos are relatively common in family 1 encapsulins [32,35,44]. However, the encapsulation of secondary cargo proteins in natural systems has only been shown for *M. xanthus* [30], in which the core cargo EncB and secondary cargos EncC and EncD encapsulate in the same encapsulin (EncA) nanocompartment. In *Mycobacterium tuberculosis*, the core cargo is a DyP peroxidase (Mt-Dyp). Two additional secondary cargo proteins were identified by the presence of a characteristic C-terminal CLP, Mt-BrfB (a bacterioferritin) and FolB (a 7,8-dihydroneopterin aldolase involved in folate metabolism) [76]. Each of these three cargos has independent antioxidant activity and is encapsulated in the nanocompartment when coexpressed with the encapsulin gene in *E. coli* [76]. In *M. tuberculosis* cells grown in standard laboratory conditions, however, only nanocompartments with the core cargo Mt-DyP have been identified [77]; results are similar for *M. smegmatis* [54].

All characterized cargo proteins are homo-oligomers, and the size and oligomeric state of the cargo proteins largely determines cargo stoichiometry within the compartment. In *B. linens*, with a T = 1 encapsulin system, loading of the cargo DyP is limited to one hexamer, a trimer of dimers, per nanocompartment [53,78], which results in a cargo:encapsulin stoichiometry of 1:10. The nanocompartment size could accommodate larger quantities of DyP monomers, but the dimensions and shape of the hexameric complex limits loading to one cargo complex per compartment. The *M. smegmatis* DyP-loaded encapsulin, a T = 1 particle with two DyP hexamers, has a stoichiometry of 1:5 [51]. EncFtn cargo forms decameric complexes arranged as pentamers of dimers [36,69]. Due to high cargo occupancy in *T. maritima*, the electron density corresponding to the CLP sequence of the EncFtn cargo was clearly identified in specific depressions on the interior penton surface of the encapsulin shell [6]. The relatively small size of dimeric EncFtn allows for unimpeded binding of 12 decameric complexes to the 12 pentons of the nanocompartment, resulting in a 2:1 stoichiometry [69]. In contrast, the EncHo system contains four of these similar EncFtn decamers in a tetrahedral arrangement that results in a 2:3 ratio [52].

The multiple contacts established by multimeric cargo CLP with the encapsulin could increase avidity of this interaction and allow greater encapsulation specificity even at low individual affinities [6]. Robust loading is nonetheless observed for monomeric heterologous proteins [50,53], although expression levels of heterologous cargo tend to be artificially high. Oligomerization might have a role in the correct positioning of assembled complexes inside the nanocompartment [6,36,53,61,69]. In the EncTm system, the disposition of the five shell-bound CLP of the EncFtn decamer in the T = 1 shell penton aligns the central ring of the decamer with the pore at the fivefold symmetry axis of the encapsulin shell [6]; this alignment facilitates iron entry from the pore to the EncFtn active site [69]. In the EncHo system, however, there is a symmetry mismatch between the four decameric EncFtn cargos and the icosahedral encapsulin shell. Decamers are offset from the interior encapsulin surface, and only two decamers align at the fivefold symmetry axes [52].

### 2.4. Effect of Encapsulin on Cargo Protein Function

Encapsulin nanocontainers are very resistant structures able to withstand a wide range of pH, temperature, and protease treatments [33,53,79], which is also extended to the encapsulated proteins. Encapsulation stabilizes the encapsulated heterologous enzymes, with an increase in their thermal stability that prolongs enzyme activity [50]. For example, the DyP peroxidase from *Saccharomonospora viridis* loses activity in ~30 min at 40 °C; when packaged inside the EncMh compartment; however, activity increases in the first few hours and decreases significantly only after 25 h [50]. This stabilization has been attributed to the molecular crowding effect [80], which suggests that the numerous protein–protein interactions inside the nanocompartments prevent irreversible unfolding and aggregation of the cargo protein [50,81].

Pores in the nanocompartment regulate the molecular flux through the shell by size and by charge [6,53,81,82], and constitute one of the key parameters in controlling encapsulated protein activity [83]. Their role as a molecular sieve is thought to assist the flux of correct substrates [36,49], preventing undesired reactions. The pores, thus, impose strong size and/or charge restrictions that lead to low enzyme activity if substrates [53,63] or cofactors [50] cannot diffuse adequately through the pores. Recent studies in the EncHo system show dynamic behavior of the major fivefold pores, in which the pores open via movement of the A-domain. The open and closed conformations range from 9 to 24 Å in diameter, respectively [52].

Native encapsulated enzymes have coevolved with encapsulin to function inside the nanocompartment. In general, their activity is enhanced compared with that of enzymes assayed outside the encapsulin shell. When encapsulated, the *S. elongatus* cysteine desulfurase shows a sevenfold increase in K_cat_ [31], for example, and the *Rhodococcus jostii* DyP peroxidase has eightfold higher activity towards lignin [74]. For the EncFtn proteins, ferroxidase activity is also higher inside the nanocompartment [69]. These examples indicate that the long evolutionary history of cargo and encapsulin nanocontainer led to the development of unique nanomachines with solutions tailored to maximize enzyme/nanocompartment synergies.

### 2.5. Physiological Role of Encapsulins

The diversity of the cargo activities in the encapsulins suggests that this compartmentalization strategy has evolved to fulfill diverse physiological roles. The multi-cargo encapsulin system of *M. xanthus* has been characterized in detail [31,84,85]. Through their ferroxidase activity, EncB and EncC can oxidize, mineralize, and sequester large amounts of iron and phosphorus [30,86,87,88]. EncA is essential for fruiting body formation [85], a starvation response that leads to sporulation [89]. The assembly of nanocompartments induced during starvation protects the cells from death due to oxidative stress caused by peroxide exposure. These findings indicated that together with ferritins, *M. xanthus* encapsulin acts as a secondary iron storage system induced by starvation to temporarily sequester iron and phosphorous as an antioxidant response [30]. Encapsulin deletion mutants of *M. xanthus* are unable to transform between the two natural phases of the organism [84].

The *M. tuberculosis* encapsulin gene Cfp29 is necessary for its growth in mice [90], and it encodes an immunodominant T cell antigen in mice and in humans [91]. The encapsulin core operon contains Cfp29 and DypB, a DyP-type peroxidase [76] that is encapsulated in Cfp29 nanocompartments [54,77]. The encapsulin system is needed to defend the bacteria from oxidative stress at low pH in a fatty acid-rich environment, reminiscent of the phagolysosome [92]. Mutants unable to express Cfp29 and DypB survive poorly in murine bone marrow-derived macrophages and are more susceptible to treatment with the antibiotic pyrazinamide [77].

In addition to fungi, several bacteria can break down lignin using ligninolytic enzymes such as DyP, laccase, and bifunctional catalase [93]. Given its great abundance and renewability, lignocellulosic biomass has important biotechnological potential for sustainable development. Bacterial DyP have activity toward large anthraquinone-based dyes and phenolic lignin model compounds, but their mode of oxidation remains unknown [67]. The bacterium *R. jostii* can metabolize nitrate lignin and its dypB gene is essential for this activity [94]. The nitrate lignin substrate is larger than the pores of the T = 1 compartment, and the encapsulated DyPB has ~8-fold greater activity for polymeric lignin than the naked enzyme. Partial disassembly of the encapsulin nanocompartment was suggested to localize DyPB close to the lignin surface, which increases its activity [74]. The DyP-type enzymes themselves catalyze the oxidation of substrates that, due to their large size, are unable to enter the enzyme active site [67]. The catalysis of these substrates was explained by electron transfer from the active site via a long-range electron transfer pathway to residues at the enzyme surface for oxidation [95,96]. An alternative hypothesis for lignin degrading activity of encapsulated DyP involves prolongation of the DyP long-range electron transfer mechanism to the encapsulin nanocompartment surface.

Several encapsulin systems have been characterized in heterologous hosts, mainly in *E. coli.* The IMEF systems are described in spore-forming Firmicutes, the majority of which do not have genes for the known primary iron storage systems, ferritin and bacterioferritins. When expressed in *E. coli*, these encapsulin systems form ~42 nm-diameter T = 4 capsids with exceptionally large iron storage capacity, leading to the suggestion that IMEF systems act as a primary iron homeostasis mechanism [49]. The hemerythrin-containing family 1 encapsulin systems encapsulate their hemerythrin cargo protein when expressed in *E. coli* and protect host bacteria from oxidative and nitrosative stress [35].

The EncSe system is implicated in the sulfur starvation response [31]. The genes of the encapsulin monomer and its cysteine desulfurase cargo are both upregulated *in S. elongatus* during sulfur starvation. When expressed in *E. coli*, the loaded encapsulin complex uses free L-cysteine as a substrate. Whether the sulfide from the cysteine remains within the compartment, which would act as a storage cage for sulfur, or it is transferred to the cellular sulfur pathway remains to be elucidated.

## 3. The Encapsulin Toolbox

Although four encapsulin families have been described so far, only family 1 systems have been used for biotechnological applications. In this section we offer an overview of the different strategies, modifications and improvements used to engineer the natural systems into useful nanomachines. In the following sections, “encapsulins” will thus refer to family 1 encapsulin systems.

### 3.1. Use of Encapsulin Systems for Nanotechnological Applications

Although the EncTm system is the most extended encapsulin among the numerous biotechnological applications, many other systems from various bacteria are being incorporated as promising nanoplatforms. The different systems used in biotechnological applications are summarized in Table 1.

### 3.2. Encapsulin Expression and Purification

In general, after codon optimization, encapsulins are efficiently expressed in *E. coli*, in some cases with exceptional yields of up to 1 g/L culture [50]. Robust expression and cargo loading are also reported in other expression systems, such as yeast [97], insect cells [98], mammalian cells [86,88,99], *Drosophila*, and mice [88].

>Classical purification protocols that are applied to viral particles and virus-like particles, consisting on differential centrifugation followed by polishing steps using size exclusion chromatography (SEC), are described in detail for *B. linens* and *T. maritima* encapsulins expressed in *E. coli* [62]. For *T. maritima*, an initial heat precipitation step is efficient for removing nucleic acid contamination [100]. Insertion of a tag (G5H6G5) in an exposed loop after amino acid 138 increases thermostability of the nanocompartment up to 90 °C, which facilitates heat precipitation and allows for metal affinity purification (IMAC) [100,101,102,103]. Encapsulins are generally recovered from the cleared culture lysate by precipitation with polyethylene glycol, heat denaturation, or ammonium sulfate precipitation. Encapsulins are usually further purified by SEC or ion-exchange chromatography, with a final SEC polishing step. For some applications, high resolution SEC has been deemed essential [82].

Tags for affinity chromatography purification inserted in the exposed encapsulin C-terminus facilitate faster purification procedures; Strep-tag II and FLAG tags have been successfully used for this purpose [86,88,99].

In mammalian cells, encapsulins can be directed through the exocytic route by N-terminal fusion with secretory signal peptides; this facilitates direct purification of assembled nanocompartments from supernatants [24,88].

### 3.3. Shell Engineering

The encapsulin shell is quite tolerant to genetic manipulation and very stable against chemical modifications; numerous alterations have hence been made in the shell to improve its natural characteristics and/or to add new functions.

Biocompatibility of encapsulin nanocompartments can be increased by PEGylation, the coating of the nanoparticle surface with polyethylene glycol [104,105]. This is a safe procedure that increases bloodstream retention time and evades immune system surveillance [106]. The natural resistance of encapsulin containers to proteases can also be enhanced by covalent attachment of *Ecballium elaterium* trypsin inhibitor II knottin to the external surface [107].

The high catalytical activity of encapsulated enzymes requires adaptation between pore characteristics and metabolite size and charge [63,83]. Two studies analyzed the influence of pore size and charge on mass transport [81,82] by systematically mutating the loop region that delineates the pores at the fivefold axis of the EncTm nanocompartment. They generated structural libraries of nanocompartments with pore variants and analyzed the effect of pore size and charge on nanocompartment structure and on small molecule flux through the nanocompartment shell. The mutant Δ9Gly2, which generates the largest pore size (~11 Å) [81], showed contradictory results, either with mass transport increased by sevenfold [81] or with minimal differences in the ion flux kinetics [82]. In an unrelated study, the Δ9Gly2 EncTm mutant showed a fivefold increase in the relative performance of a nanoreactor-catalyzed enzyme cascade when compared to wild type EncTm [83]. These results underscore the difficulties inherent to accurately quantifying flux into protein cages and warrant further investigation. Alternatively, these conflicting results might be related to the highly dynamic pore structure, which is not particularly discriminatory for small molecules, as shown for the EncHo system [52].

Although TP-mediated cargo loading of heterologous proteins has been used widely in family 1 encapsulin systems, greater understanding is needed of the molecular mechanisms involved in this process, to allow design of more complex multi-component systems. In an initial approach, the TP-shell interactions on T = 1 (EncTm) and T = 3 (EncMx) encapsulin systems have been analyzed and novel TP sequences were designed for both systems [34]. Using mNeoGreen as cargo protein, fusion with these TP sequences led to reduced cargo loading, an outcome similar to that predicted by a computer model.

Nanocompartments are functionalized, in addition to cargo loading, by inserting modifications in the protein shell. In family 1 encapsulins, the protein shell amino and carboxy termini are displayed at the inner and outer surfaces of the nanocompartment, respectively, allowing simple functionalization strategies. In the case of EncTm, insertions are well tolerated in several shell protein loops, which allows further possibilities for inserting multiple functional domains into the same nanocontainer [100,102,108].

The possibilities of rapid shell functionalization have been greatly expanded with the SpyTag/SpyCatcher system [109], a technology is based on the capacity of the short SpyTag peptide (13 amino acids) to form a spontaneous intermolecular isopeptide bond with the SpyCatcher protein (12.3 kDa). The reaction is very specific and the protein domains involved are functional when fused to other proteins. Insertion of the SpyTag domain in the EncTm loop on position 138 is well tolerated and leads to nanocompartments with 60 SpyTag copies on the surface [110]. Simply mixing the purified nanocompartment with proteins fused to a SpyCatcher domain covers the nanocompartment surface with the recombinant protein. A similar strategy has been reported using a SpyCatcher fusion on the C terminus of the EncTm encapsulins [83].

## 4. Encapsulin-Based Nanotechnological Applications

The characteristics that make encapsulin systems very efficient natural nanoreactors also render them useful tools for biological research and for the development of a variety of engineering applications. These features are (1) encapsulins are very simple modular systems, as a single shell protein is able to self-assemble into relatively large icosahedral shell architectures of ~22 nm (T = 1), ~32 nm (T = 3), and ~42 nm (T = 4); (2) the nanocompartments are highly monodisperse, mechanically rigid, pH-resistant, and temperature-stable; (3) numerous pores regulate the flux of metabolites, by charge and size, through the protein shell; (4) there are natural mechanisms for efficient functionalization of the exterior surface by genetic fusion with the shell protein, and of the inner volume using short CLP for cargo protein loading; and (5) they are genetically encoded and very tolerant of genetic manipulation.

### 4.1. Encapsulins as Nanoreactors

**Figure 4:**
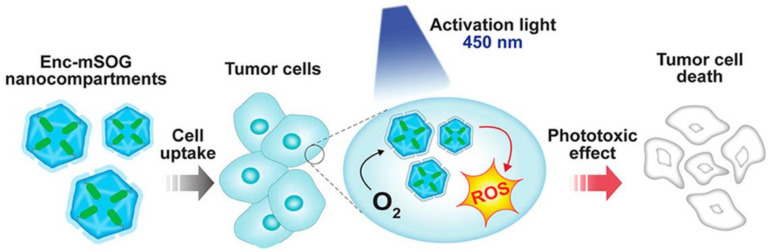
An EncTm nanoreactor with a reversed oxidative stress response. Overview for delivery, activation, and phototoxic effect of miniSOG-loaded EncTm nanocompartments. Via endocytosis, tumor cells take up photosensitizing EncTm-miniSOG nanocompartments; following blue-light excitation, the nanocompartments photoconvert intracellular O_2_ to cytotoxic ^1^O_2_ (singlet oxygen) that induces tumor cell death. Reprinted with permission from [103]. Copyright 2021 American Chemical Society.

Family 1 encapsulins are in essence natural nanoreactors with a simple, CLP-based mechanism for internalizing enzymes and other cargo proteins; this has fostered the engineering of nanocompartments as nanoreactors using a variety of enzymes [50,63,83,88,97,103,111]. Confinement of enzymatic reactions within encapsulin shells is particularly suited to overcoming problems associated with toxicity of intermediate products and competition by metabolic activities [97]. In an innovative study, the EncTm system was converted into a light-responsive nanoreactor able to induce production of reactive oxygen species (ROS) after irradiation with a blue light laser [103]. A modified version of EncTm [100] was loaded with the mini singlet oxygen generator protein (miniSOG) by fusing a CLP to its C-terminal end. When irradiated with blue light, miniSOG generated singlet oxygen (^1^O_2_), a highly reactive ROS [112,113]. MiniSOG-loaded nanocompartments were able to produce ~2-fold more singlet oxygen than free miniSOG (Figure 4). The capacity of the nanoreactor to exert a light-activated phototoxic effect on cells was evaluated in vitro in a lung cancer cell model, which showed a ~34% reduction in cell viability.

**Figure 5:**
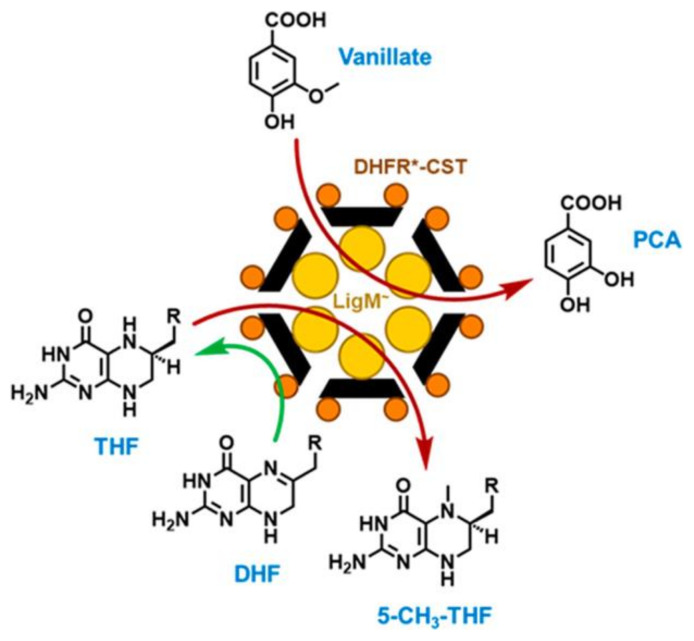
A two-enzyme EncTm-based nanoreactor. EncTm-scaffolded metabolon design. Surface-immobilized DHFR enzymes (orange spheres) generate THF in situ from DHF (green reaction arrow); a THF-dependent demethylase enzyme (LigM, yellow spheres) uses THF for subsequent reaction with vanillate (lignin-derived aryl substrate) inside the EncTm nanocompartment (red reaction arrows). Reprinted with permission from [83]. Copyright 2021 American Chemical Society.

A recent report described construction of a complex EncTm-based nanoreactor with functional domains fused in- and outside the protein shell [83]. This multienzyme nanoreactor is based on the *Sphingomonas paucimobilis* aryl-O-demethylase (LigM) [114], which is incorporated within the nanocontainer with a CLP bound to its C-terminal end, and on the *E. coli* dihydrofolate reductase (DHFR), which is covalently immobilized on the exterior using the SpyTag/SpyCatcher system. For this, a SpyCatcher domain is inserted in the EncTm C terminus, and DHFR is tagged with a SpyTag domain on its C terminus. The final nanoreactor contains 60 DHFR molecules and ~30 LigM enzymes (Figure 5). The activity of these nanoreactors was ~5 times slower than that of the free enzymes in solution, probably due to the narrow pores of the nanocompartment, which was verified when the pores at the fivefold axes of the nanocompartment were enlarged using the ∆9Gly2 mutation [81]. In these conditions, the nanoreactor activity was as efficient as the free enzymes in solution.

### 4.2. Encapsulins as Targeted Delivery Systems and Nanovaccine Platfforms

Targeting biomolecules to a specific cell type is of utmost importance for increasing the therapeutic value of a treatment and reducing its potential complications and is the focus of intense research [115]. Several studies show that encapsulin can serve as an effective platform for targeted drug delivery.

Fusion of the hepatocellular carcinoma cell targeting peptide SP94 [116] to residue 138 in the exposed loop of EncTm allows specific interaction, followed by internalization of the modified nanocompartment in HepG2 tumor cells [100]. When the modified nanocompartments are additionally functionalized by chemical crosslinking with the acid-sensitive prodrug aldoxorubicin, internalization of these functionalized EncTm results in controlled doxorubicin release into the acidic environment of the tumor cells. This treatment reduces cell viability similar to treatment with free doxorubicin.

Protein-based nanoparticles have very repetitive surfaces that render surface-displayed epitopes strongly immunogenic [117], and surface proteins on the encapsulin shell produce potent humoral immune responses. When fused to the EncTm C terminus, the D_123_ domain of the Epstein–Barr virus major envelope glycoprotein gp350 produces nanocontainers that display 60 copies of D_123_ on the outer shell surface. Mice and non-human primates immunized with these nanoparticles show potent virus-neutralizing immune responses [24]. In addition, a specific antibody response can be induced against internally loaded proteins. Mice immunized with GFP-loaded EncTm nanocompartments and modified to display the M2e epitope of the influenza A virus on their outer surface show a specific immune response to both polypeptides [108]. Peptides on the EncTm encapsulin surface also induce strong, specific cellular responses [118].

### 4.3. Encapsulins as Genetically Encodable Materials for Biological Imaging

The background activity generated by unencapsulated cargo is an inherent problem of the use of encapsulins as a genetically encoded system. One strategy that can overcome this problem is the split protein system that uses bimolecular complementation [119]. In this method, two fragments of a fluorescent protein (or enzyme), which do not produce a signal unless closely associated, are targeted simultaneously to the nanocompartment, and their close proximity reconstitutes their function. This approach was used successfully in yeast with the split-Venus fluorescent protein [97,120], in which the two components of the N-Ven and C-Ven system were tagged with a CLP specific for the EncMx nanocompartment. Results were similar using the PAmCherry1 split fluorescent protein system and the split luciferase NanoLuc [88].

Signal contrast is also increased by exploiting the protection against protease degradation offered by encapsulation. In this approach, heterologous protein cargos are tagged simultaneously with CLP and with strong destabilizing signals that target them for degradation by the proteasomal machinery. After expression, non-encapsulated cargo proteins are rapidly degraded, which removes background and greatly increases the signal-to-background ratio for the encapsulated fluorescent proteins. This method was assayed in yeast using the mNeonGreen fluorescent protein with the EncMx system [97]. Similar systems have been developed using EncMx [88] or EncQt [99] in human cells.

Whereas the penetration depth of fluorescence signals is limited to approximately a millimeter [121], sound waves (used in photoacoustic imaging) and magnetic fields (used in magnetic resonance imaging; MRI) are minimally attenuated by biological tissues and offer a viable alternative for deep imaging in vivo [122]. The EncMx system has been engineered to act as a reporter for photoacoustic tomography (PT) in conjunction with the strong photoabsorbance of melanin [88]. The cytoplasmic tyrosinase of *Bacillus megatherium* [123] was thus tagged for encapsulation by fusion to the C terminus of an *M. xanthus* natural cargo protein, and human cells expressing the encapsulin and the cargo protein showed melanin production and strong contrast in photoacoustic images.

**Figure 6:**
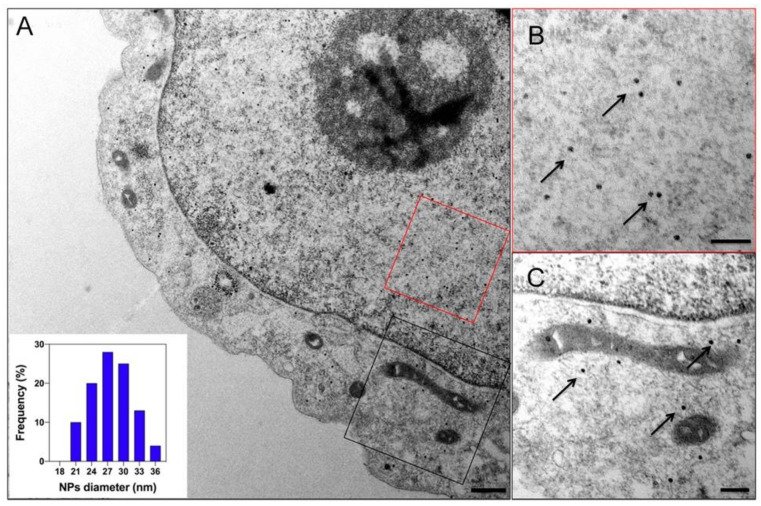
Iron oxide nanoparticle formation within EncQt nanocompartments in human cells. (**A**) Electron microscopy thin section image of HepG2 cells transiently expressing EncQt with its natural ferroxidase IMEF and the iron transporter Zip14. Bar, 500 nm. The inset shows the size distribution of iron oxide cores inside the encapsulin nanocontainers. (**B**,**C**) The close-up views show representative areas in the nucleus ((**B**), red rectangle) and cytosol ((**C**), black rectangle)). Arrows indicate individual iron-containing nanoparticles. Bar, 200 nm. Reprinted from [99].

EncMx and EncQt systems, which are able to biomineralize large iron cores are viable alternatives as genetically encoded MRI reporters [88,99]. Transient expression in human cells of EncMx with its natural ferritin-like cargo produced intense signals in MRI analysis [88]. The large iron core in EncMx and EncQT also offers superior characteristics as a genetically encoded fiduciary markers for cryo-EM and reporter for TEM [86,88,99] (Figure 6). Following low expression of the iron transporter Zip14 [124], both EncQt and EncMx encapsulin systems have strong biomineralization activity, in these conditions accumulating ~35,000 and ~19,000 iron atoms per shell, respectively.

### 4.4. Encapsulin-Based Metallic Nanoparticles

The bottom-up approaches for biometallic nanoparticle synthesis are of great interest, as they allow for precise control of particle morphology, have lower energy requirements, avoid use of toxic materials, and require less-expensive materials [125]. Constrained synthesis of metallic nanoparticles using protein nanocontainers has several additional benefits, including production of monodisperse particles of defined size and the possibility of generating nanoparticles with multiple functions via genetic or chemical modification of the protein shell [111].

**Figure 7:**
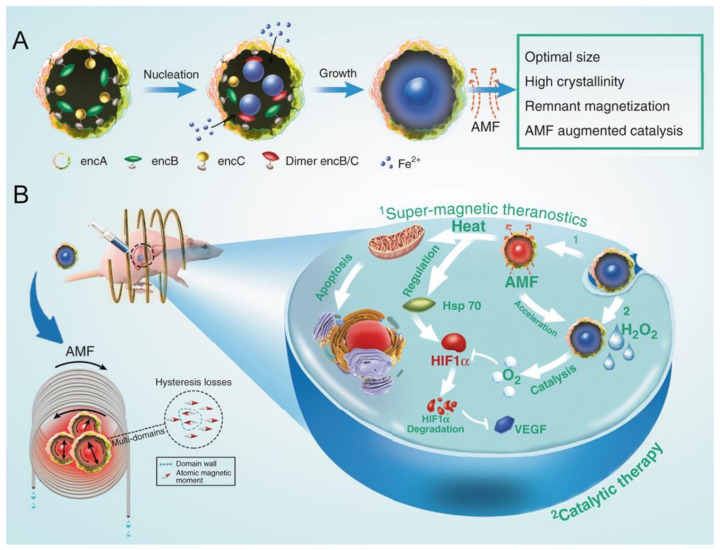
Engineering of encapsulin-produced magnetic iron oxide nanoparticles (eMIONs) for magneto-catalytic therapy. (**A**) Production of eMIONs by the in vitro biomineralization of EncMx containing EncB and EncC cargo proteins. (**B**) eMIONs accumulated in tumors suppressed tumor growth. In the presence of an alternative magnetic field (AMF), eMIONs induce magnetic hyperthermia (left arrow) and have enhanced catalase-like activity (right arrow). Reprinted from [87].

Encapsulin systems that biomineralize iron naturally provide an efficient means of generating magnetic nanoparticles, with promising applications in magnetic hyperthermia therapy (MHT) [87]. Clinical trials of MHT have been approved for different types of cancer using superparamagnetic iron oxide nanoparticles [126]. The EncMx system was used to generate magnetic nanoparticles, which are monodisperse, resistant to extreme pH and protease digestion, and stable when exposed to blood and serum. These eMIONs (encapsulin-produced magnetic iron oxide nanoparticles) efficiently absorb magnetic energy that results in a pronounced temperature increase in vitro and in vivo when exposed to an alternative magnetic field. In addition, eMIONs can decompose H_2_O_2_ into O_2_, thus inducing specific tumor cell toxicity and apoptosis due to the elevated H_2_O_2_ concentration in cancer cells. Given the high penetration of magnetic fields, eMIONs are a promising therapeutic agent for the treatment of deep solid tumors (Figure 7).

The EncTm system has been used as a platform to synthesize size-constrained silver nanoparticles [111]. The sequence that encodes the silver-precipitating peptide AG4 [127] was fused to the N terminus of the encapsulin gene. In the engineered compartment, the inner surface is thus lined with 60 copies of the AG4 peptide. After mineralization with AgNO_3_, the protein shell was removed to liberate monodisperse Ag nanoparticles of an average 13.5 nm diameter. Silver ions and colloidal silver, including Ag nanoparticles, are known bactericidal and bacteriostatic agents [128]. Disk diffusion assays showed that bacterial pathogenic strains were susceptible to protein-coated and shell-free Ag nanoparticles.

Gold nanoparticles have also been encapsulated into EncTm nanocontainers [75]. The nanoparticles were functionalized with two components; first, with a stabilizing ligand shell (MUTAB) [129] and second, with a small number of EncTm CLP sequences, followed by encapsulation by mixing with encapsulin protomers. This strategy considerably expands the range of possible cargo for encapsulin systems and opens up new possibilities for functionalization of metallic particles via the genetic and chemical modification of the encapsulin shell container.

## 5. Future Prospects

Although research in encapsulin systems is a relatively new field, it has already produced several promising biotechnological applications. The encapsulin systems that biomineralize large metal cores appear to be efficient alternatives to ferritin, one of the most widely used protein nanocages in nanotechnology. Significant progress has been made in the discovery and description of new encapsulin systems. Experimental characterization of model systems for different operon types will undoubtedly provide better comprehension of the range of possibilities offered by the encapsulin scaffold. Some information obtained through computer analysis suggests novel cage architectures and modifications of the encapsulin shell that could lead to innovative uses.

Initial steps have been taken to understand the physical characteristics that govern reactor efficiency. Continued work in this area will provide the knowledge needed to engineer pore size and charge to regulate mass transport as a step towards the construction of more efficient systems. Further research to decipher the mechanisms that direct protein loading into the nanocompartments will allow control over the total protein loaded and the relative stoichiometry of multi-cargo systems, key parameters in engineering more complex reactions.

Research efforts to gather more information on the physiological function of encapsulins will also uncover the naturally evolved strategies for deployment and control of encapsulin systems; there are interesting early results regarding natural mechanisms for post-translational regulation of nanocompartment assembly/degradation.

A remarkable number of strategies for functionalizing the encapsulin scaffold have already been described; combined with the large library of natural cargo enzyme activities and with researcher ingenuity, they promise a productive future for research on encapsulin systems.

## Figures and Tables

**Table 1:** Encapsulin systems used for biotechnological applications.

Name	Application	References
*Brevibacterium linens M18*	Biological imaging	[62,79]
	Shell improvement	[107]
*Mixococcus xanthus*	Biological imaging	[88]
	Nanoreactor engineering	[88,97]
	Hyperthermia therapy	[87]
*Quasibacillus thermotolerans*	Biological imaging	[86,99]
*Mycolicibacterium hassiacum*	Nanoreactor engineering	[50]
*Thermotoga maritima*	Targeted delivery	[100,101,110]
	Vaccine development	[24,108,118]
	Nanoreactor engineering	[83,103]
	Microbial peptide synthesis	[102]
	Biometallic nanoparticle synthesis	[111]
*Rhodococcus erythropolis N771*	Shell improvement	[104,105]
	Nanoreactor engineering	[63]
